# Phosphorylation of NR2B NMDA subunits by protein kinase C in arcuate nucleus contributes to inflammatory pain in rats

**DOI:** 10.1038/srep15945

**Published:** 2015-10-30

**Authors:** Fan Bu, Huiyu Tian, Shan Gong, Qi Zhu, Guang-Yin Xu, Jin Tao, Xinghong Jiang

**Affiliations:** 1 Key Laboratory of Pain Basic Research and Clinical Therapy, Department of Physiology & Neurobiology, Medical College of Soochow University, Suzhou 215123, P.R. China; 2 Jiangsu Key Laboratory of Translational Research and Therapy for Neuro-Psycho-Diseases, Institute of Neuroscience, Soochow University, Suzhou 215123, P.R. China

## Abstract

The arcuate nucleus (ARC) of the hypothalamus plays a key role in pain processing. Although it is well known that inhibition of NMDA receptor (NMDAR) in ARC attenuates hyperalgesia induced by peripheral inflammation, the underlying mechanism of NMDAR activation in ARC remains unclear. Protein kinase C (PKC) is involved in several signalling cascades activated in physiological and pathological conditions. Therefore, we hypothesised that upregulation of PKC activates NMDARs in the ARC, thus contributing to inflammatory hyperalgesia. Intra-ARC injection of chelerythrine (CC), a specific PKC inhibitor, attenuated complete Freund’s adjuvant (CFA) induced thermal and mechanical hyperalgesia in a dose-dependent manner. *In vivo* extracellular recordings showed that microelectrophoresis of CC or MK-801 (a NMDAR antagonist) significantly reduced the enhancement of spontaneous discharges and pain-evoked discharges of ARC neurons. In addition, CFA injection greatly enhanced the expression of total and phosphorylated PKC_γ_ in the ARC. Interestingly, CFA injection also remarkably elevated the level of phosphorylated NR2B (Tyr1472) without affecting the expression of total NR2B. Importantly, intra-ARC injection of CC reversed the upregulation of phosphorylated NR2B subunits in the ARC. Taken together, peripheral inflammation leads to an activation of NMDARs mediated by PKC activation in the ARC, thus producing thermal and mechanical hyperalgesia.

Chronic pain, a major health issue all over the world, is caused by tissue or nerve injuries under different pathophysiological conditions. Previous studies showed that the arcuate nucleus (ARC) of the mediobasal hypothalamus is one of the critical structures in the modulation of nociception and pain^1^,^2^,^3^,^4^,^5^. Persistent peripheral nociceptive stimuli result in arcuate amplification of pain (central sensitisation)^6^,^7^,^8^, which can be seen as an increase in the magnitude of responses to a defined sensory stimulus at the level of neurons. Diverse molecules and receptors, like the ionotropic glutamate NMDA receptors (NMDARs), modulate neuronal excitability^9^,^10^,^11^,^12^. Our previous studies showed that the expression of the NR2B subunit, a functional subunit of the NMDAR, increases in the rat ARC after hindpaw injection of complete Freund’s Adjuvant (CFA)^13^. In addition, intra-ARC injection of MK-801 attenuates hyperalgesia induced by neuropathic pain^14^. However, the underlying mechanisms for the activation of NMDARs in the ARC remain unclear.

Protein kinase C (PKC), a phospholipid-dependent serine/threonine kinase, plays an important role in signal transduction pathways^15^. PKC activation involves phosphorylation, and translocation from the cytosol to the binding domains at cell membranes^16^,^17^,^18^,^19^,^20^. In particular, PKC is involved in many aspects of cellular sensitisation, including modulation of channel conductivity by phosphorylation, increased trafficking of receptors to the cell membrane, and release of excitatory neurotransmitters^9^,^21^,^22^,^23^. There are at least twelve isoforms of PKC. PKCγ is thought to play an important role in nociceptive processing^21^,^24^,^25^. *In vitro* direct phosphorylation may be a mechanism by which PKC regulates the function of NMDARs^26^. Additionally, PKC indirectly potentiates NMDAR responses by activation of the tyrosine kinase signalling cascade in CA1 pyramidal neurons of the hippocampus^27^.

Thus, these observations raise two possibilities; 1) PKC in the ARC plays a role in inflammatory pain processing in the ARC; 2) PKC activation in the ARC leads to the phosphorylation of NMDARs following peripheral inflammation. In this study, three measures were used to answer these questions. First, behavioural tests were performed to compare the effect of a PKC antagonist in normal saline- (NS) and CFA-injected rats. *In vivo* extracellular recordings were employed to measure the spontaneous and evoked responses of ARC neurons. Western blot analysis was performed to detect PKC and NR2B subunit expression in CFA-induced peripheral inflammation. Our results showed that peripheral inflammation led to a significant upregulation of PKC expression and phosphorylation of NR2B subunits in the ARC. Inhibition of PKC activity suppressed NR2B phosphorylation and thus attenuated the mechanical and thermal hyperalgesia. Collectively, these data suggest that phosphorylation of NR2B-containing NMDARs medicated by PKC in the ARC contributes to inflammatory pain in rats, thus identifying a potential molecular target for the treatment of inflammatory pain.

## Methods

### Induction of inflammatory pain

Experiments were performed in adult male Sprague-Dawley (SD) rats weighing 200 ~ 250 g. Rats were housed in cages with free access to food and water, and maintained in a climate-controlled room on a 12 h: 12 h day/night cycle. All experiments were approved by the Institutional Animal Care and Use Committee of the Medical College of Soochow University and were in accordance with the ethical standards of the International Association for the Study of Pain. Every effort was made to minimise both the number of animals used and the animal suffering. To induce inflammatory pain, CFA (100 μl, Sigma) was injected subcutaneously into the left hindpaw, as described previously^13^. CFA injection led to an obvious tissue inflammation of the hindpaw characterised by erythema, oedema, and hyperpathia^28^. Age-matched male SD rats injected with NS (0.9%, 100 μl) were used as controls. All experiments were conducted 7 days after NS or CFA injection, when the symptoms of persistent inflammatory pain were evident.

### Surgery

The rat was initially anesthetised by chloral hydrate (4%, 1 ml/100 g). The trachea was cannulated to allow mechanical ventilation with room air. The ventral surgical approach to expose the rat hypothalamus was performed by retracting the hemisectioned mandibles laterally, drawing the tongue caudally, dividing the soft palate, and removing the sphenoid bone with a dental drill, as described previously^29^. After the operation, the rat was mounted supinely in a stereotaxic apparatus. The rectal temperature was maintained at 37.0 ± 0.5 °C via an under-body heating pad. A pair of bipolar silver hook electrode was placed under the sciatic nerve for electrical stimulation. The exposed hypothalamus was covered with warm (~37°C) saline solution (0.9%). The rat was artificially ventilated with a small animal ventilator. Pyrolaxon (1.2%, 0.5 ml/100 g) and chloral hydrate were used simultaneously during the whole experiment, and maintained within the range indicated.

### Electrophysiological recordings and identification of ARC neurons

For extracellular recording and microiontophoresis, three-barrel micropipettes were used^30^, which were made with MP-87 microelectrode puller with a tip diameter of around 4 ~ 8 μm. One barrel of the three-micropipette was used to record extracellular unit discharges in ARC following stereotaxic coordinates: 0.2 ~ 1.0 mm caudal to the front age of pituitary, 0 ~ 0.5 mm lateral from midline and depth of 0.2 ~ 0.8 mm from the brain surface. The electrode was placed into ARC with the microelectrode pusher. The electrical signal was amplified and displayed on an oscilloscope, and then sent to a computer system. Neurons were included whose spike configuration remained constant and could be clearly discriminated from baseline throughout the experiment. ARC neurons were identified by its background activity and/or responses to noxious mechanical stimulus (pinching a fold of skin of the left hindpaw with toothed forceps) and electrical stimulus (parameters: 15 V considered to noxious stimulus, delay 0.1 ms, wave width 0.5 ms, interval 1 ms, 2 pulses included in one series impulse). Extracellularly recorded single-unit activities (action potentials) were recorded by Chart 5.0 software.

Every recording site was marked by electrophoretic ejection of pontamine sky blue from the recording electrode (−100 μA, 10 min). After the last recording, rat was sacrificed by overdose of chloral hydrate, and the brain was removed and soaked in paraformaldehyde (10%) for a week. The brain was then frozen and cut into 50 μm slices for subsequent histological study. The recording sites were histologically verified. Data from experiments in which histological analysis revealed recording sites outside ARC were not included in the present study.

### Drug application

#### Microelectrophoresis

Three-barrel (microfilament-filled) glass electrode was used for microelectrophoresis (tip diameter, 4 ~ 8 μm). The recording barrel (resistance, 8 ~ 10 MΩ) was filled with 1% pontamine sky blue in 0.5 M NaAc solution. The peripheral barrels were infused with the indicated solutions for microelectrophoresis. The drug barrel was filled with solutions of chelerythrine (CC; 0.4 or 2 mM, dissolved in saline, pH 4.0, Sigma) or MK-801 (1 or 3 mM, dissolved in saline, pH 4.0, Sigma). The remaining barrel was filled with NaCl (165 mM, pH 7.0). Microelectrodes were slowly inserted, using a microelectrode manipulator, until the tip of the microelectrode reached the ARC of the hypothalamus. After the identification of ARC neurons, drugs were ejected. All drugs were ejected with a positive current of 20–30 nA using a JL-H2001 microelectrophoresis apparatus (Jialong Educational Instrument Factory, Shanghai, China), and a stagnation negative current of 3 ~ 5 nA was applied between ejections to prevent drug leakage. During administration, noxious electrical stimuli were applied every 5 s to observe the drug effect on noxious responses of pain excitatory neurons (PENs) in the ARC. We confirmed that the injection of NS did not alter neuronal firing. To eliminate the possible electrical effects caused by microiontophoresis, data were discarded if current ejection from pipettes elicited any changes in the spontaneous discharge of the recorded neurons.

#### Implantation of intracerebral guide cannula and drug injection

The rat was anesthetised by chloral hydrate (4%, 1 ml/100 g) and mounted on a stereotaxic instrument. A sterilised stainless-steel guide cannula (20 gauge) was positioned 2.8 mm dorsal to the ARC [AP: −4.0, L: unilateral 0.5, V: 9.8 mm; AP, anterior (+) or posterior (−) to Bregma; L, lateral to midline; V, ventral to the surface of skull], as described by Paxinos and Watson^30^, and fixed to the skull by dental acrylic. Rats were allowed to recover from the surgery for at least 3 days. An injection needle (26 gauge) was directly inserted into the guide cannula for drug administration. The injection needle, which extended 2.8 mm beyond the tip of the guide cannula, was connected by a catheter to a microsyringe. One microliter of the solution was thereafter infused into the ARC over 2 minutes. Solutions for intra-ARC injection were prepared with sterilised NS (0.9%); each volume of 1 μl contained 0.5, 1, or 2 nmol of CC. Sterilised NS (0.9%) served as the control. The injection solution was slowly delivered into the ARC as described in our previous study^13^. The injection needle was left in place for at least 30 s after each injection before removal. This procedure prevented the injection solution from spreading dorsally along the injector tract. At the end of each experiment, rats were sacrificed by overdose of chloral hydrate, and the heads were fixed in 4% formalin for 24 h with the injection tube *in situ* before sectioning. The tip of the injection needles was verified to locate in the ARC.

### Behavioral studies

All behavioral studies were carried out in a quiet room from 9:00 am to 11:00 am. To minimize the stress induced by handling and measurements, rats were accustomed to the test condition for 5 days before the experiment. On the day of experiment, rats were allowed to habituate to the environment for 30 min before experiments began. The thermal paw-withdrawal latency (TWL) and mechanical paw-withdrawal threshold (MWT) were used to determine hyperalgesia during subsequent measurements (tested at 0, 10, 20, 30, 40, 50, 60 and 90 min after drug injection).

#### Thermal paw-withdrawal latency (TWL) testing

For measurement of TWL, the tail flick unit (Ugo Basile) was used^31^. Rats were gently held, and the left hindpaw pad was put over the flush-mounted window containing the heat source set at 50 °C. The hindpaw withdrawal latency was measured in seconds and defined as the time taken by the rat to remove its left hindpaw from the heat source. The cut-off point was set at 10 seconds to prevent potential tissue damage^32^.

#### Mechanical paw-withdrawal threshold (MWT) testing

von Frey filaments (VFFs) were employed to measure mechanical hyperalgesia. A series of calibrated VFFs (0.4 ~ 25.0 g) were applied perpendicularly to the plantar surface of the hindpaw with sufficient force to bend filaments for 60 s or until it withdrew. In the absence of a response, filament of next greater force was applied. In the presence of a response, filament of next lower force was applied. To avoid injury during experiments, cutoff strength of VFF was 25.0 g. The tactile stimulus producing a 50% likelihood of withdrawal was determined by means of the "up-down" calculating method, as described in detail previously^33^,^34^. Each test was repeated 2 ~ 3 times at ~2 minute interval, and the average value was used as the force to induce a withdrawal response.

### Western blot analysis

One week after NS or CFA injection, rats were deeply anesthetized and sacrificed. ARC region was cut out quickly. Three ARC slices (500 μm thick) were obtained from each rat and moved into ice-cold artificial cerebrospinal fluid. The ARC slices were homogenized with the phosphatases and proteases inhibitors. The homogenate was centrifuged at 13,000 g for 10 minutes at 4°C and supernatant was used for western blot analysis. The protein concentration in the homogenate was measured using a bicinchoninic acid (BCA) kit. Proteins were separated by SDS-PAGE using Criterion XT Precast 10% Bis-Tris gels (Bio-Rad, Hercules, CA) and electrotransferred onto PVDF membranes (Invitrogen) in standard transfer buffer (0.25 M Tris, 0.192 M glycine, 10% vol/vol methanol, pH = 8.3) at room temperature for 3 hours. After the membranes were blocked with 5% non-fat milk in Tris-buffered saline and 0.1% Tween-20 (TBST) for 1 hour, bound proteins were exposed to anti-PKCγ (1:1000, mouse monoclonal antibody, Santa Cruz) antibodies, anti-PKCγ-pThr514 (1:3000, Rabbit monoclonal antibody, Cell Signaling) antibodies, anti-NR2B (1:1000, rabbit polyclonal antibody, Millipore) antibodies, anti-NR2B-pTyr1472 (1:1000, rabbit polyclonal antibody, Calbiochem) antibodies, and anti-β-actin (1:2000, rabbit monoclonal antibody, Cell Signaling) antibodies at 4 °C over night. After extensive washing in TBST, a 1:8000 dilution of goat anti-rabbit or goat anti-mouse horseradish peroxidase secondary antibody (Bioworld Co. Ltd) was used as appropriate and incubated for 1.5 hours at room temperature. After extensive washing, signals were detected with Western Lightning ECL and quantified relative to β-actin control by densitometry on Image J software.

### Statistical analysis

The neuronal responses to noxious stimulus were measured and expressed as spikes per second (Hz) by subtracting any background activity in the 1 s preceding the stimulus from the total activity in the 1 s after the stimulus. The effects of drugs were determined using the following equation^35^: effects = (firing frequency before treatment − firing frequency after treatment)/firing frequency before treatment × 100%. Behavior data were analyzed using one-way ANOVA with *post hoc* Bonferroni comparison or two-sample *t* test as appropriate. The proportion of PENS in ARC was analyzed using χ^2^ test. Electrophysiological data were analyzed using two-sample *t* test. Western blot data were analyzed using Mann-Whitney test or Kruskal-Wallis following the Dunn’s test as appropriate. All data were presented as mean ± S.E.M. p < 0.05 was considered statistically significant.

## Results

### Inhibition of PKC signalling in the ARC attenuates thermal and mechanical nociceptive responses

**Figure 1:**
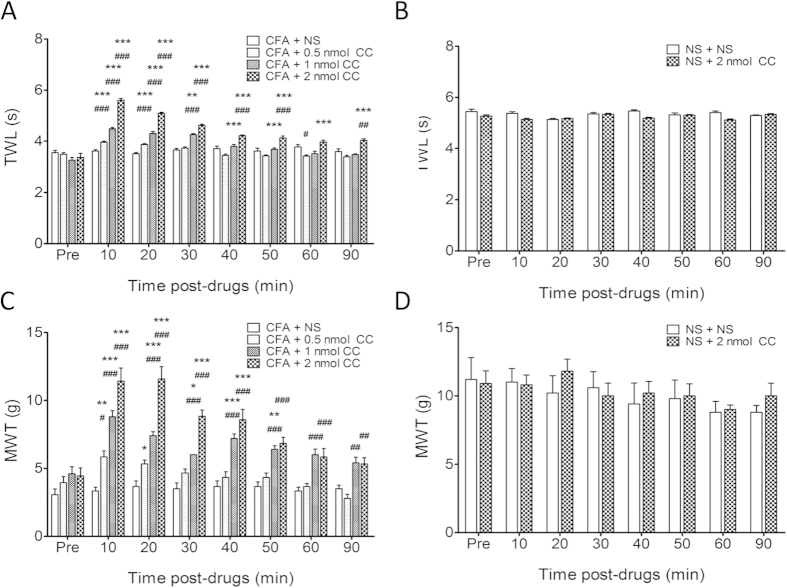
Inhibition of PKC signalling by chelerythrine (CC) attenuates pain behaviour in CFA-injected rats. (**A**) CC decreased thermal hyperalgesia in CFA rats. Latency of thermal withdrawal responses (TWL) increased 10 min after injection and lasted for more than 30 min. CC at the dose of 2 nmol had the maximal analgesic effect on paw withdrawal latency in CFA rats. (**B**) CC at the dose of 2 nmol failed to produce any effect on paw withdrawal latency of age-matched control rats. (**C**) CC decreased mechanical hyperalgesia in CFA rats. Mechanical paw withdrawal threshold (MWT) increased 10 min after microinjection and lasted for more than 30 min. CC at the dose of 2 nmol produced the maximal analgesic effect on MWT in CFA rats. (**D**) CC at the dose of 2 nmol failed to cause any effect on MWT of age-matched control rats. *p < 0.05, **p < 0.01, ***p < 0.001 *vs.* pre-drug; ^#^*p* < 0.05, ^##^*p* < 0.01, ^###^*p* < 0.01 *vs.* CFA + NS.

We first examined the effect of intra-ARC injection of the PKC inhibitor CC on nociceptive responses to thermal and mechanical stimulation in CFA-induced inflammatory rats. CFA rats were divided into four groups, each of which received intra-ARC injections of CC at 0.5 nmol (n = 12), 1 nmol (n = 10), or 2 nmol (n = 12), or 1 μl of 0.9% NS (n = 12) as a control. As shown in Fig. 1, both paw withdrawal latency to thermal stimulation (TWL, Fig. 1A) (For 4 levels between subjects (treatment), F values were as follows: 10 min: 280.4, 20 min: 188.2, 30 min: 89.74, 40 min: 22.21, 50 min: 15.45, 60 min: 11.30, 90 min: 16.04. For 8 levels within subjects (time), F values were: CFA + 0.5 nmol CC: 22.42, CFA + 1 nmol CC: 42.25, CFA + 2 nmol CC: 78.23, CFA + NS: 1.016) and paw withdrawal threshold to mechanical stimulation (MWL, Fig. 1C) (For 4 levels between subjects (treatment), F values were: 10 min: 34.10, 20 min: 39.59, 30 min: 41.90, 40 min: 20.45, 50 min: 18.43, 60 min: 11.11, 90 min: 12.86. For 8 levels within subjects (time), F values were: CFA + 0.5 nmol CC: 7.215, CFA + 1 nmol CC: 12.40, CFA + 2 nmol CC: 15.86, CFA + NS: 0.35) increased significantly after intra-ARC injection of CC in a dose dependent manner (*p < 0.05, **p < 0.01, ***p < 0.001 vs. PRE; ^#^*p* < 0.05, ^##^*p* < 0.01, ^###^*p* < 0.001 vs. CFA + NS). The anti-nociceptive effects of CC at all three doses were seen at 10 min when the first measurement was performed, and at the maximal dose of 2 nmol, the effects persisted through the whole observation period (90 minutes). In contrast, NS injection failed to produce any inhibitory effect in rats with hindpaw inflammation. In addition, microinjection of CC at 2 nmol into the ARC had no marked effect on nociceptive responses to thermal (Fig. 1B, *n* = 10) and mechanical stimulation (Fig. 1D, *n* = 10) in age-matched non-inflammatory controls. Collectively, these results indicate that PKC in the ARC is involved in the pathogenesis of hyperalgesia following peripheral inflammation.

### Inhibition of PKC signalling attenuates spontaneous and evoked responses of ARC neurons to noxious stimulus

**Figure 2:**
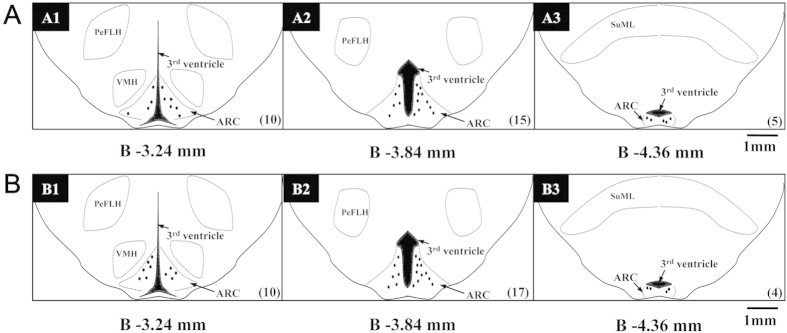
Histological verification of recorded PENs in the ARC. The standard diagrams show coronal sections at three levels posterior to Bregma (A1, B1: −3.3 mm; A2, B2: −3.8 mm; A3, B3: −4.3 mm). Coronal sections are based on atlas plates from Paxinos and Watson (1998). Symbols (black dots) show the positions of the tip of the microelectrophoresis probe in the ARC. (**A**) In representative coronal sections, recorded PENs (*n* = 30) in the ARC from control rats are indicated. (**B**) In represe*n*tative coronal sections, recorded PENs (*n* = 31) in the ARC from CFA rats are indicated. ARC: arcuate nucleus; 3 V: 3rd ventricle. The numbers in parentheses under each figure indicate the number of neurons recorded in the ARC.

**Figure 3:**
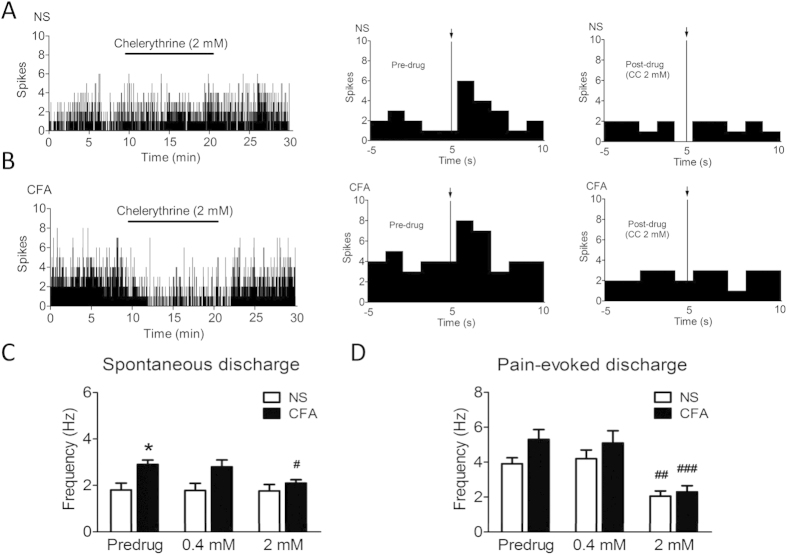
Inhibition of PKC signalling by chelerythrine (CC) suppresses the excitability of ARC neurons. (**A**) A representative example of the spontaneous discharges of one PEN from an NS- (top) and CFA-injected rat (bottom). CC (2 mM, +20 ~ 30 nA, 15 min) was used. (**B**) Bar graph shows the effects of CC on the spontaneous discharges of PENs in the ARC of NS- and CFA-injected rats. (**C**) A representative example of the noxious-electrical-stimulus evoked discharges of one PEN each from NS- (top) and CFA-injected rats (bottom). CC (2 mM, +20 ~ 30 nA, 15 min) inhibited the pain-evoked discharges of PEN in NS or CFA rat. Arrows show the noxious electrical stimuli. (**D**) Bar graph showing that CC suppressed the pain-evoked discharges of PENs both from NS- and CFA-injected rats. *p < 0.05 vs. NS; ^#^*p* < 0.01, ^##^*p* < 0.001 *vs.* pre-drug.

We next determined whether inhibition of PKC signalling attenuates ARC neuronal activity. Based on the responses to peripheral noxious stimuli, ARC neurons are categorised into three types: pain excitatory neurons (PENs), pain inhibitory neurons (PINs), and non-responsive to somesthetic stimulus neurons (noSOMs)^13^. We focused on PENs because they represented the class of ARC neurons that are consistently sensitised to afferent inputs in the CFA pain model^31^. The locations for the extracellular single unit recording of PENs in ARC are shown in Fig. 2. In ARC, 67 neurons were recorded for the NS group and 30 out of 67 (44.9%) were PENs (Fig. 2A). 56 neurons were recorded for the CFA group and 31 out of 56 (55.2%) were PENs (Fig. 2B). The proportion of PENs in ARC was significantly increased after CFA injection.(44.9%*vs.* 55.2%) (*p* < 0.05), which was in agreement with out previous work^31^. Additionally, the spontaneous and pain-evoked discharges of PENs increased significantly in CFA rats as compared with that in age-matched control rats (Fig. 3A,B, *p* < 0.05). Following microiontophoresis of CC at a dose of 2 mM in the ARC for 15 min, the spontaneous discharges were markedly reduced in CFA rats (*n* = 10, Fig. 3A, bottom). The average frequency of spontaneous discharges before and after CC application was 2.76 ± 0.26 Hz and 2.07 ± 0.13 Hz after CC application, respectively (*n* = 10, Fig. 3A bottom, and 3B, ^#^*p* < 0.05, compared with pre-drug). Moreover, the pain-evoked discharges were also greatly inhibited in CFA injected rats (Fig. 3C,D). The average frequency of pain-evoked discharges in CFA rats before and after CC application was 5.27 ± 0.33 Hz and 2.39 ± 0.36 Hz, respectively (*n* = 10). Thus, application of CC in the ARC by microiontophoresis significantly inhibited pain-evoked discharges in CFA rats (Fig. 3C,D, ^###^*p* < 0.001, compared with pre-drug). Application of CC at 0.4 mM did not produce any effect on spontaneous and pain-evoked responses of ARC neurons both in NS- and CFA-treated rats. Of note, application of CC at the dose of 2 mM did not significantly alter the number of spontaneous discharges of ARC neurons in age-matched NS-injected rats (Fig. 3A upper, and 3B, *n* = 8). Interestingly, application of CC at 2 mM substantially inhibited the number of evoked responses of ARC neurons in age-matched control rats (Fig. 3D, *n* = 8, ^##^*p* < 0.01, compared with pre-drug). The average frequency of evoked discharges in NS-injected rats before and after CC application was 3.91 ± 0.36 Hz and 2.07 ± 0.33 Hz, respectively. Collectively, these data suggest that PKC in the ARC participates in the regulation of excitability of PENs following peripheral inflammation.

### CFA injection upregulates the expression of PKC_γ_ in the ARC

**Figure 4:**
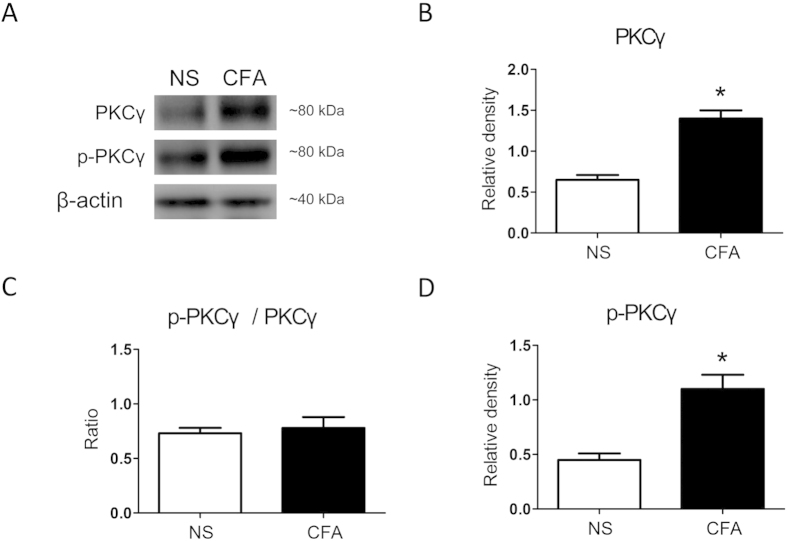
Upregulation of PKCγ expression in the ARC induced by peripheral inflammation. (**A**) Representative immunoblots against anti-PKC_γ_, anti-p-PKC_γ_, and anti-β-actin antibodies (*n* = 3 for each group). (**B**) The relative level of PKC_**γ**_ protein was significantly higher in the CFA group than in the NS group (*p < 0.05, *n* = 3 for each group). (**C**) The relative level of p-PKC_**γ**_ protein was markedly higher in the CFA group than in the NS group (*p < 0.05 vs. NS, *n* = 3 for each group). (**D**) The ratio of p-PKC_γ_**/**PKC_**γ**_ remained unchanged after CFA injection. All experiments were repeated at least twice.

We further examined the expression of PKC_γ_, an important subtype of PKC, which has been reported to be involved in pain processing^32^,^33^,^34^. CFA injection markedly enhanced the expression of PKC_γ_ (Fig. 4A,B, *p < 0.05) (also see full length blots in Fig. S1). The expression of PKC_γ_ in the ARC relative to the internal control was 0.61 ± 0.76 in the NS group (n = 3) and 1.46 ± 0.18 in the CFA group (*n* = 3). In addition, the level of phosphorylated PKC_γ_ (p-PKC_γ_) in the ARC was measured. The relative value of p-PKC_γ_ in the ARC was 0.42 ± 0.027 in the NS group (*n* = 3) and 1.09 ± 0.09 in the CFA group (*n* = 3). CFA injection significantly enhanced the expression of p-PKC_γ_ (Fig. 4A,C, *p < 0.05). However, the ratio of p-PKC_γ_ to total PKC_γ_ was unaltered (Fig. 4D), which suggested that an increase in p-PKC_γ_ expression did not exceed the increase in total PKC_γ_ expression after CFA-induced peripheral inflammation.

### Inhibition of PKC signalling reduces phosphorylation of NR2B subunits in ARC

**Figure 5:**
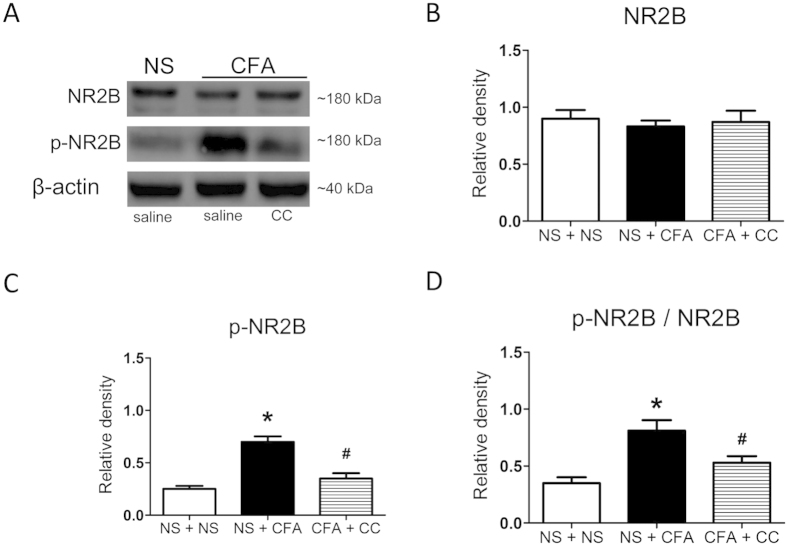
Inhibition of PKC signalling by chelerythrine (CC) decreases the expression of p-NR2B (Tyr1472) in the ARC. (**A**) Representative immunoblots of ARC against anti-p-NR2B (Tyr1472) and anti-NR2B antibodies from NS- and CFA-injected rats (*n* = 3 for each group). All experiments were repeated at least twice. (**B**) The relative level of total-NR2B proteins was u*n*altered after CFA injection. Similarly, CC treatment did not have any significant effect on total NR2B expression (*p* > 0.05, *n* = 3 for each group). All experiments were repeated at least twice. (**C**) The level of p-NR2B (Tyr1472) was significantly higher in the CFA group than in the NS group. CC treatment markedly suppressed the expression of p-NR2B (Tyr1472) in CFA group as compared with the NS group. (**D**) The ratio of p-NR2B**/**NR2B was higher in the CFA group as compared with the NS-injected rats. CC treatment significantly reduced the ratio. *p < 0.05 vs. NS + NS; ^#^*p* < 0.05 vs. CFA + NS, *n* = 3 for each group. All experiments were repeated at least twice.

We next investigated whether PKC signalling is linked to the upregulation of NR2B subunit of the NMDAR following peripheral inflammation. The level of NR2B expression was examined at 10 min after intra-ARC injection of 2 nmol CC or NS. Consistent with our previous report^13^, CFA injection did not significantly alter the level of total NR2B expression (Fig. 5A,B) (also see full length blots in Fig. S2). However, CFA injection led to a significant upregulation of phosphorylated NR2B (p-NR2B) expression (Fig. 5A,C, *p < 0.05). Consequently, the ratio of p-NR2B to total NR2B was significantly higher after CFA injection (Fig. 5D, *p < 0.05), indicating that the increase in p-NR2B expression exceeds the increase in total NR2B expression after CFA injection. We then examined changes in the expression of total and p-NR2B after intra-ARC injection of CC in CFA rats. Application of CC greatly reduced the expression of p-NR2B (Fig. 5A,C, ^#^*p* < 0.05), while the level of total NR2B remained unaltered (Fig. 5A,B). The ratio of p-NR2B to total NR2B after CC injection was significantly reduced when compared with the CFA + NS group (Fig. 5D, ^#^*p* < 0.05). Together, these findings suggested that the decrease in the level of p-NR2B subunit by inhibition of PKC signalling contributes to reduction in inflammatory pain responses.

### Blockade of NMDAR activity suppresses spontaneous and evoked firing of ARC neurons

**Figure 6:**
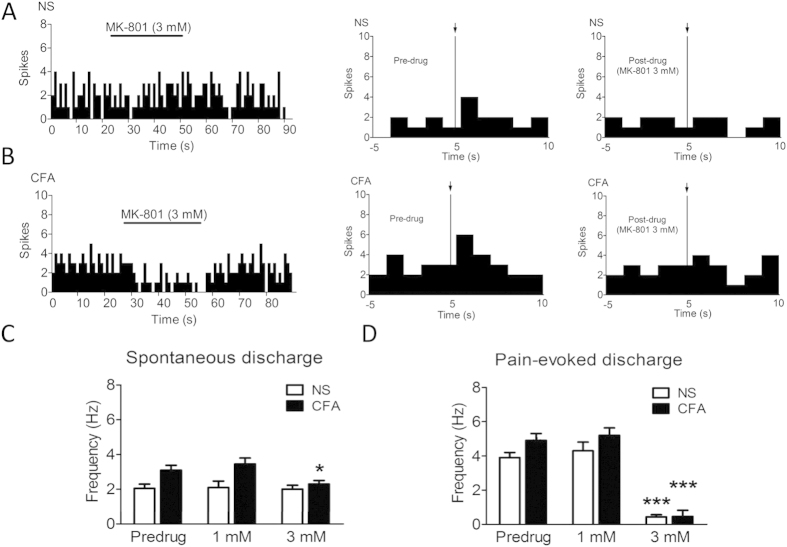
Blockade of NMDA receptor activity by MK-801 suppresses the excitability of ARC neurons. (**A**) Administration of MK-801 (3 mM, +20 ~ 30 nA, 30 s) failed to alter the spontaneous discharges of the ARC neuron from an NS-treated rat (top), but significantly inhibited the spontaneous discharges of a PEN from CFA-injected rat (bottom). (**B**) Bar graph showing the effects of MK-801 on the spontaneous discharges of PENs from NS- and CFA-injected rats. (**C**) Administration of MK-801 (3 mM, +20 ~ 30 nA, 30 s) significantly inhibited the pain-evoked discharges of a PEN in NS or CFA rat. Arrows show noxious electrical stimulus. (**D**) Bar graph showing that administration of MK-801 significantly reduced the pain-evoked discharge frequency of PENs in NS- and CFA-injected rats. *p < 0.001 *vs.* pre-drug. *n* = 11 cells in each group.

We next determined the role of the arcuate NMDAR in peripheral inflammatory pain. Two different concentrations of MK-801, a NMDAR antagonist, were delivered to PENs in the ARC by microiontophoresis. After administration of MK-801 at 3 mM for 30 s, the frequency of spontaneous discharges of PENs in CFA rats (n = 11) markedly decreased (Fig. 6A,B, 1.91 ± 0.13 Hz *vs.* 3.12 ± 0.26 Hz, *p* < 0.001, compared with pre-drug). Similarly, administration of MK-801 at 3 mM significantly reduced the frequency of pain-evoked discharges of PENs in CFA rats (*n* = 11) (Fig. 6C,D, 0.51 ± 0.36 Hz *vs.* 4.70 ± 0.48 Hz, *p* < 0.001, compared with pre-drug; *t = 3.623, NS ***t = 5.392, CFA ***t = 6.155). MK-801 at the dose of 1 mM failed to produce any effect on spontaneous discharges and pain-evoked responses of PEN in age-matched control rats (n = 11) (Fig. 6A,B, *p* > 0.05, compared with pre-drug). However, administration of 3 mM MK-801 significantly reduced the frequency of pain-evoked discharges of PENs in age-matched control rats (n = 11) (Fig. 6A,B, 0.36 ± 0.20 Hz *vs.* 3.84 ± 0.35 Hz, *p* < 0.001, compared with pre-drug). Therefore, these results suggested that NMDARs are involved in the change in excitability of PENs in the ARC of inflamed rats.

## Discussion

In this study, it was demonstrated for the first time that PKC plays an important role in the phosphorylation of NR2B subunits of NMDARs in ARC of rats with peripheral inflammation. Our previous study showed that peripheral inflammation enhanced ARC neuronal activity, which was blocked by an NMDAR antagonist microinjected into the ARC^7^, and that arcuate Src activation contributed to tyrosine phosphorylation of NR2B subunit of NMDARs^13^. In the present study, we provide new evidence to demonstrate that NMDA activity is also regulated by PKC in the ARC following peripheral inflammation. Application of PKC inhibitor CC not only significantly attenuated pain hypersensitivity, but also reversed the upregulation of tyrosine-phosphorylated NR2B protein expression in rats with peripheral inflammation. Furthermore, both CC and MK-801 inhibited the spontaneous and pain-evoked discharges of ARC neurons in CFA-injected rats *in vivo*. Together with our previous studies^7^,^13^, these data suggest that sensitisation of arcuate PKC and NR2B is involved in pain hypersensitivity following peripheral inflammation.

There is a growing body of evidence showing that the PKC cascade is involved in the initiation of persistent nociceptive sensitisation^36^,^37^,^38^,^39^ and that the PKC subtypes play different roles in different intracellular signalling pathways under pathophysiological conditions^15^,^18^. An interesting finding in the present study is that the expression of PKC_γ_ and *p*-PKC_γ_ was enhanced in the ARC following peripheral inflammation. PKC_γ_ expressed in the diencephalon, midbrain, and pons-medulla regions is involved in antinociception in mice^25^. In contrast, chronic constriction nerve injury in rats is substantially attenuated in PKCγ knock-out animals^32^. We assessed the involvement of PKC_γ_ in inflammatory pain in ARC, and showed that PKC_γ_ is detectable in ARC by immunoblots, which is consistent with previous reports^40^. Interestingly, CFA injection significantly enhanced the expression of total PKCγ, p-PKCγ, and p-NR2B (Tyr1472). However, the ratio of p-PKCγ/total PKC did not change while the ratio of p-NR2B/total NR2B was greatly enhanced. This indicates that the regulatory mechanisms are different for these two molecules. PKC participates in pain processing through its enhanced expression of total proteins while NR2B is involved in pain signalling via its phosphorylation. Since p-PKCγ is significantly enhanced after peripheral inflammation, the enhanced *p*-PKCγ may contribute to the phosphorylation of NR2B, thus causing pain hypersensitivity. Of note, CC chloride used in the present study is an inhibitor of both novel and classic PKC isoforms^18^,^41^. After CFA treatment, the expression of both total PKCγ and *p*-PKCγ increased significantly. Parenthetically, we cannot exclude the possibility of the involvement of other subtypes of PKC in the ARC in nociceptive sensitisation following peripheral inflammation. Further studies are needed to clarify whether other subtypes of PKC in ARC are involved in the maintenance of inflammatory pain.

Another important finding is that PKC regulates neuronal excitability through the activation of NMDARs in ARC following peripheral inflammation. It is known that ARC exhibits large clusters of *β*-endorphinergic neurons, and is one of the critical structures in antinociception. However, for years, a growing body of evidence has shown that ARC may play a dual role in pain processing: nociceptive descending inhibition and descending facilitation^1^,^2^,^3^,^4^,^5^,^6^,^7^,^8^,^13^,^14^. In the present study, we provided new evidence to demonstrate that ARC neurons are hyperactive following peripheral inflammation. To simplify our analysis, we adopted our previous regimen and selected the pain-evoked neurons in the present study. Our electrophysiological studies showed that peripheral inflammation led to an increase in spontaneous discharges and evoked responses of accurate PENs to peripherally applied noxious stimuli. This suggested that accurate PENs are involved in the transmission of nociceptive signal and arcuate sensitisation. In addition, application of an NMDAR antagonist and PKC inhibitor suppressed pain-evoked discharges of PENs, in a dose-dependent manner. Since the electrophysiological observations of PKC and NMDAR activity were not recorded in the same PENs, we do not have direct evidence to show that activation of PKC modulated NMDAR activity in PENs in the ARC. However, the activities of almost all PENs, which were influenced by CC, were also blocked by MK-801. Furthermore, we showed that arcuate activation of NMDARs was PKC-dependent in rats with peripheral inflammation. CC significantly inhibited the upregulation of the expression of phosphorylated NR2B in CFA-injected rats. Although we do not know whether PENs are endorphin-producing cells, the present data and previous studies^13^ suggest that PENs in the ARC are activated, and that an increase in PKC activity phosphorylates the NR2B subunits of NMDARs. To better understand the significance of these molecular and electrophysiological data, further investigation of ARC in the development and maintenance of inflammatory pain is needed. Although the detailed mechanisms by which PKC activation regulates the function of NMDARs in ARC neurons have yet to be studied, phosphorylation of the NMDAR by PKC results in an increase in channel conductance or potentiation of glutamate-gated currents manifested as an enhanced excitability of the ARC neuron^27^,^42^.

Protein phosphorylation is a major mechanism for the regulation of receptor function^43^,^44^,^45^,^46^. Phosphorylation of multiple sites on NMDARs in the cytoplasmic COOH termini of the NR1 and NR2 subunits is known to modulate NMDAR activity and affect synaptic transmission^47^,^48^,^49^. Our previous study showed that arcuate Src activation induced tyrosine phosphorylation of NR2B subunit at Tyr1472, thus contributing to inflammatory pain^13^. An increase in tyrosine phosphorylation of both NR2A and NR2B was blocked by pretreatment with the selective PKC inhibitor CC in rat hippocampal CA1^50^. In the present study, thermal and mechanical hyperalgesia were significantly attenuated 10 min after intra-ARC injection of 2 nmol CC while the expression of p-NR2B (Tyr1472) was markedly reduced in CFA-injected rats. Since inhibition of PKC lowered the NR2B tyrosine phosphorylation very quickly^12^, it is tempting to speculate that PKC-induced tyrosine phosphorylation of NR2B subunits may be an important step in the PKC-mediated potentiation of NMDAR function. However, how PKC affects the phosphorylation of NR2B in ARC under inflammatory conditions remains largely unknown^51^,^52^,^53^,^54^,^55^. Since administration of the PKC inhibitor CC did not alter the expression of p-Src (data not shown), it is unlikely that Src is involved in the phosphorylation of NR2B by PKC. Further studies are needed to determine the detailed mechanisms by which PKC_γ_ regulates phosphorylation of NR2B in the ARC during the inflammatory process.

In summary, these findings demonstrate that PKC-NMDAR coupling in PENs of the ARC is involved in arcuate nucleus sensitisation following CFA-induced peripheral inflammation. PKC plays an important role in the tyrosine phosphorylation of NR2B subunits of NMDARs. We speculate that arcuate PKC promotes the activation of NMDAR, increases the excitability of ARC neurons, and eventually causes hyperalgesia following peripheral inflammation. The signalling pathway of PKC-NMDAR needs to be studied further.

## Additional Information

**How to cite this article**: Bu, F. *et al.* Phosphorylation of NR2B NMDA subunits by protein kinase C in arcuate nucleus contributes to inflammatory pain in rats. *Sci. Rep.* **5**, 15945; doi: 10.1038/srep15945 (2015).

## Supplementary Material

Supplementary Information
