# Supplementary material for: Phosphorylation of NR2B NMDA subunits by protein kinase C in arcuate nucleus contributes to inflammatory pain in rats
Source: Sci Rep. 2015 Oct 30;5:15945. doi: 10.1038/srep15945 (PMC4626761; doi:10.1038/srep15945)
Supplement: Supplementary Information [file srep15945-s1.pdf]

Supplemental Materials

Phosphorylation of NR2B NMDA subunits by protein kinase C in arcuate nucleus contributes to inflammatory pain in rats

Fan Bu, Huiyu Tian, Shan Gong, Qi Zhu, Guang-Yin Xu, Jin Tao, Xinghong Jiang

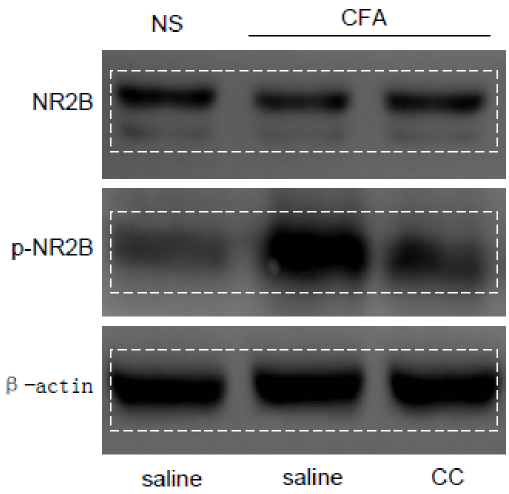

Fig. S1: Full-length blots for NR2B expression in ARC. Shown is the expanded image of western blot for NR2B against a loading control, GAPDH, presented in Fig. 4A. The Blot is representative of three experiments.

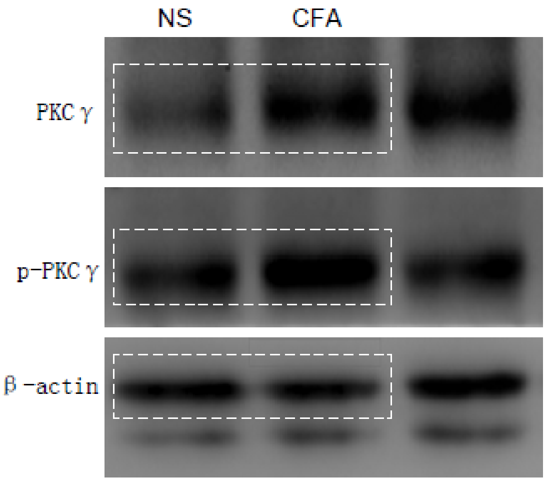

Fig. S2: Full-length blots for PKCγ expression in ARC. Shown is the expanded image of western blot for PKCγ against a loading control, GAPDH, presented in Fig. 5A. Blot is representative of three experiments.
